# Using Qualitative Research Methods to Codesign a Virtual Community of Practice for Global Oncology Early-Career Investigators

**DOI:** 10.1200/GO-25-00686

**Published:** 2026-08-27

**Authors:** Fatou Jallow, Amina Chtourou, Sabrina Epou, Mishka Kohli Cira, Kalina Duncan, Patti Gravitt, Sudha Sivaram

**Affiliations:** ^1^ Center for Global Health, US National Cancer Institute, Rockville, MD

## Abstract

**PURPOSE:**

Cancer incidence and mortality are projected to increase disproportionately in low- and middle-income countries (LMICs), highlighting the need to strengthen global cancer research capacity. Supporting early-career investigators (ECIs) is central to this effort. We conducted a qualitative study to identify barriers and facilitators to global oncology research careers among ECIs and to inform the codesign of a virtual community of practice (CoP).

**METHODS:**

We conducted semistructured in-depth interviews with ECIs participating in the US National Cancer Institute's Global Cancer Research Training Program between February and June 2023. Participants were purposively selected to represent diverse disciplines and geographic regions. Interviews explored career goals, institutional support and constraints, mentorship experiences, and training needs. Transcripts were analyzed using inductive thematic analysis, and the findings were synthesized to guide CoP design with relevant stakeholders.

**RESULTS:**

Three themes were identified: institutional capacity, mentorship, and alignment of research with local priorities. Participants described challenges related to infrastructure, protected time, and access to training. They emphasized the need for structured networking, skills development in grant writing and research methods, and platforms to support peer collaboration. These findings informed CoP design features, including competency-focused programming, cross-institutional networking, and resource-sharing mechanisms.

**CONCLUSION:**

This study describes a qualitative approach to user-centered design of a virtual CoP for ECIs in LMICs. Centering participant perspectives informed the development of a context-responsive platform for training, collaboration, and knowledge exchange.

## INTRODUCTION

In 2022, an estimated 20 million new cancer cases and 9.7 million cancer-related deaths were reported globally, with incidence and mortality projected to increase substantially by 2050.^1^ While high-income countries (HICs) will experience the greatest absolute number of cases, the largest proportional increases are expected in low- and middle-income countries (LMICs), where cancer deaths may double.^2^

**CONTEXT**

**Key Objective**
To develop a virtual community of practice (CoP) that facilitates capacity-building in global cancer research, by inviting perspectives from early-career investigators in low- and middle-income countries (LMICs) to guide CoP design and inform strategies for its implementation.
**Knowledge Generated**
By directly connecting identified needs and gaps in cancer research training shared by early career investigators to CoP design features, this approach demonstrates how qualitative insights inform user-driven program development and the importance of codesign and inclusive engagement in developing sustainable capacity-building platforms.
**Relevance**
By supporting a community of, for, and developed by early-career researchers, this strategy has the potential to enhance learning, resource sharing, and collaboration in cancer research.

Cancer epidemiology varies across regions and populations, including within the United States.^3-6^ Notably, cancers more prevalent in LMICs, such as liver cancer, are increasing in some US subpopulations.^7-9^ These trends underscore the need for global cancer research to better understand diverse risk factors, environmental exposures, and health system constraints.

**FIG 1:**
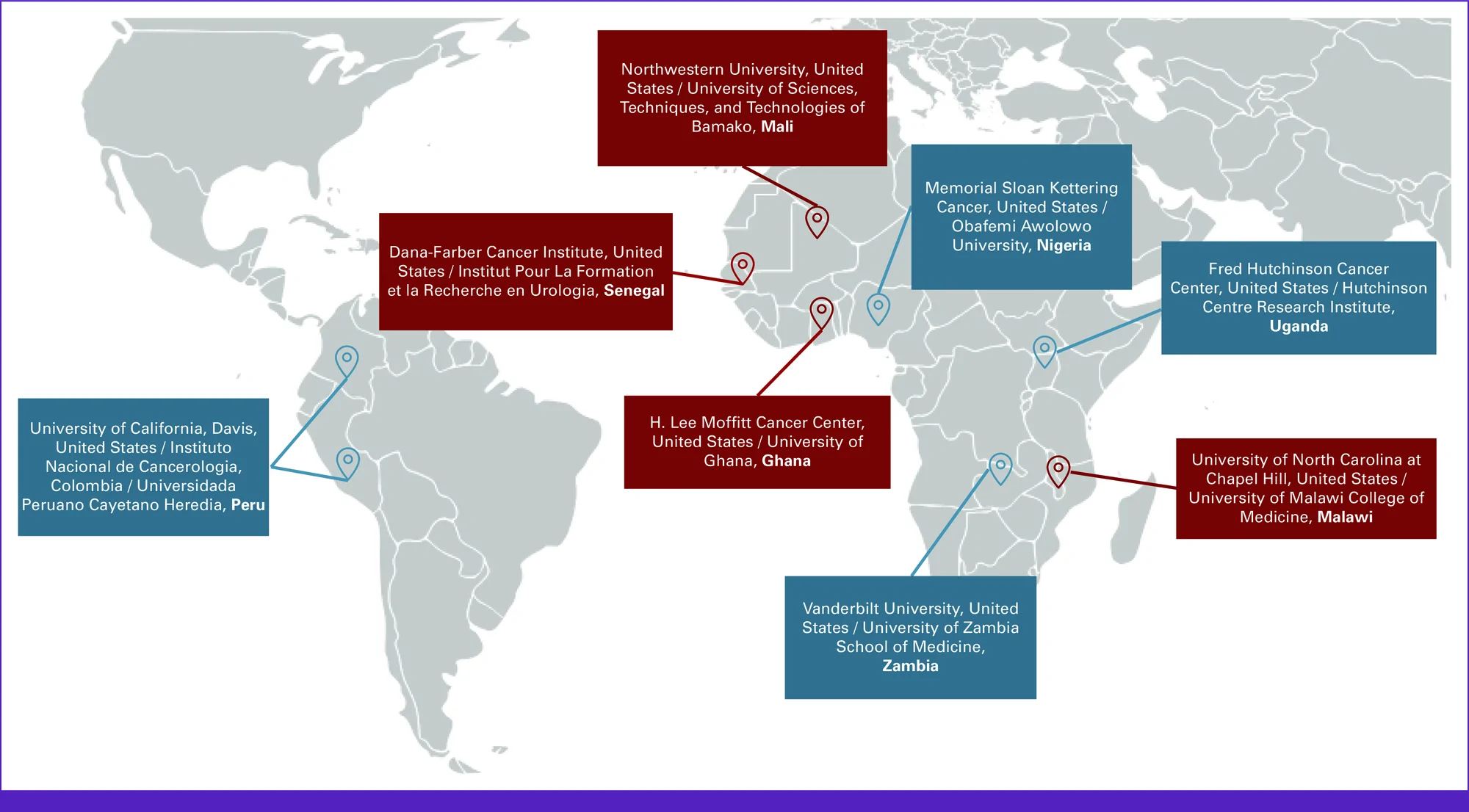
Map of NCI's Global Cancer Research Training Program with LMIC Collaborators. LMIC, low and middle-income country; NCI, National Cancer Institute.

Strengthening the global oncology workforce is essential to addressing this burden.^10,11^ Demand for training among early-career investigators (ECIs) is increasing in both the United States and LMICs, yet opportunities remain limited, particularly in resource-constrained settings.^12,13^ In response, the US National Cancer Institute (NCI) launched the Global Cancer Research Training Program (GCRTP)^14^ in 2020, awarding eight grants to support degree training, mentored research, and curriculum development (Fig 1).

To complement these training efforts and support sustained learning and collaboration across institutions, additional models that facilitate peer engagement and knowledge exchange are needed. Communities of practice (CoPs) provide a structured approach to collaborative learning and knowledge exchange.^15-22^ Defined by three components: domain, community, and practice, CoPs have been shown to enhance knowledge exchange, professional networks, and skill development.^20,23-26^ Virtual CoPs are particularly valuable in resource-limited settings, where access to centralized training may be limited, and peer exchange can support equitable participation.^27^ Evidence suggests that codesigned CoPs with end-users are more effective and sustainable.^24-26,28-31^

To inform the development of a CoP within GCRTP, we conducted a qualitative study with LMIC-based ECIs to understand their experiences navigating cancer research careers and to identify priorities for community design. Participants included pre- and postdoctoral candidates across clinical, basic, and population sciences from sub-Saharan Africa and Latin America. This study presents these findings and describes how they informed the codesign and formative development of a virtual CoP. In this study, ECIs refer to individuals from LMICs in NCI's GCRTP who are conducting research at their home institutes.

## METHODS

Methods are described in two phases: (1) Qualitative study design and (2) Codesign of the virtual community of practice (CoP).

### Qualitative Study Design

This study reflects the preimplementation phase of CoP development. We conducted a qualitative study using semistructured in-depth interviews (IDIs)^32^ to explore the experiences, aspirations, and training needs of ECIs participating in NCI's GCRTP. With IDIs, we elicited rich, individual-level insights into contextual barriers, facilitators, and expectations related to global cancer research careers.

#### Sampling and Participant Recruitment

Purposive sampling was used to identify ECIs across the eight GCRTP training sites. Principal investigators nominated participants representing diverse disciplines, training stages, and geographic regions. Twenty-nine ECIs were invited; 19 consented and completed interviews.

#### Data Collection

Interviews were conducted via videoconference (Zoom) between February and June 2023 by the lead author (F.J.) and lasted approximately 60 minutes. A semistructured interview guide (Appendix Table A1) explored career goals, institutional supports and challenges, mentorship experiences, research skill gaps, and perceptions of collaborative platforms; questions served as guiding prompts, and the interviewer used probes and follow-up questions as needed based on participants' responses.

Interviews were audio-recorded, transcribed verbatim, and supplemented with field notes. Simultaneous translation in French and Spanish was made available.

#### Ethical Considerations

The NIH Institutional Review Board determined this study to be exempt from human research. All participants provided informed consent.

#### Data Analysis

Transcripts were analyzed using Dedoose (Version 9.0.107).^33^ Two researchers (F.J. and S.S.) independently conducted inductive thematic analysis.^34^ Codes were developed iteratively, compared, and refined into a shared coding construct and then grouped into higher-order themes through discussions. An audit trail was maintained to enhance analytic rigor. Thematic sufficiency was reached when no new themes emerged.

Findings were synthesized and reviewed with stakeholders and ECIs to inform CoP design.

### Codesign of the Virtual CoP

**FIG 2:**
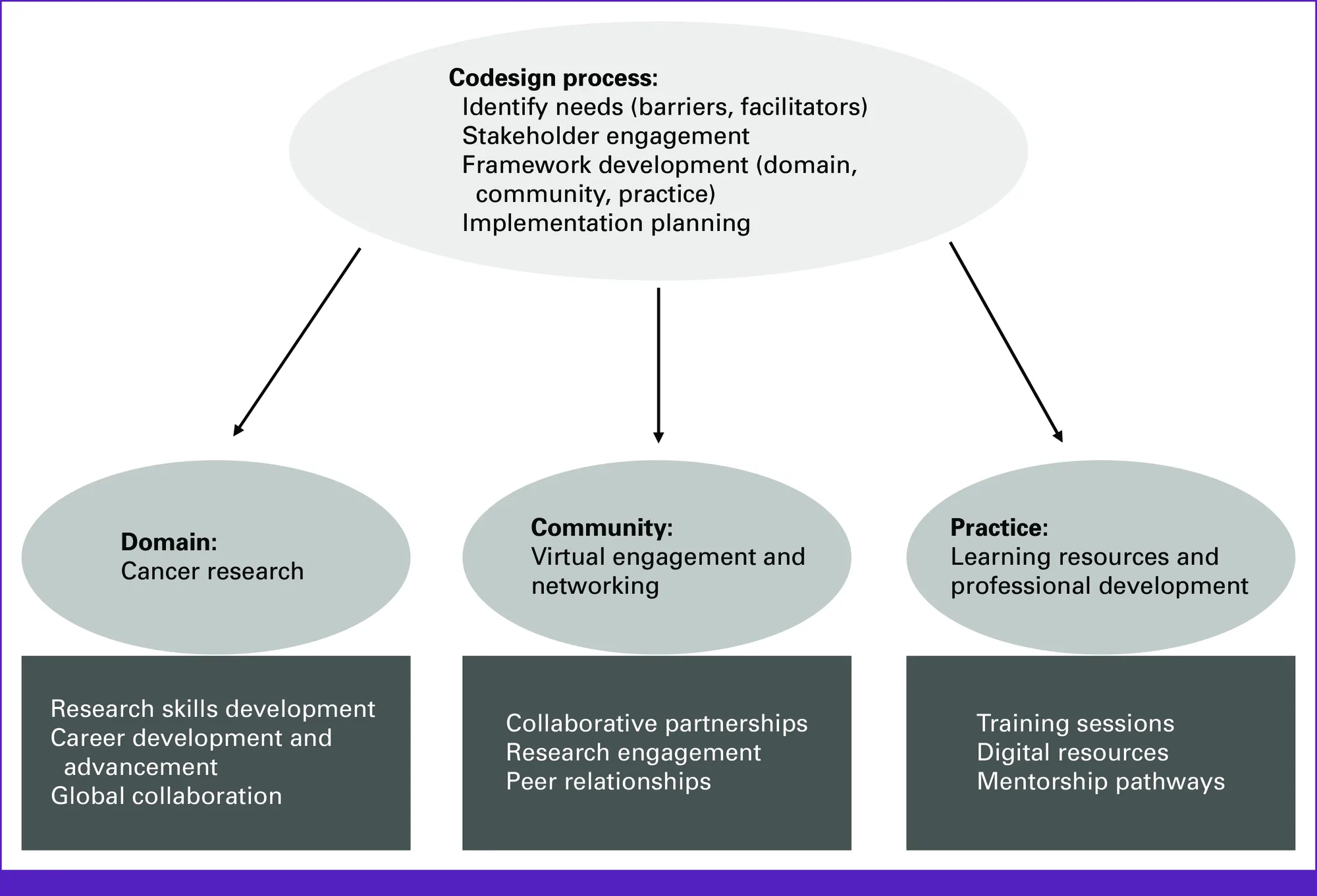
Codesign framework for a virtual community of practice in global oncology.

The CoP framework codesign process (Fig 2) began by identifying barriers and facilitators through the qualitative study described above. A core team comprising five NCI Center for Global Health (CGH) staff with experience in ECI support and CoP implementation, along with eight ECI stakeholders, who served as the initial steering committee, guided subsequent codesign steps: framework development and implementation planning. Using a participatory approach, the team translated qualitative findings into design features through a series of structured virtual meetings over 2 months.

The Results section presents the thematic findings from the IDIs and describes how these findings were applied to inform the codesign of the virtual CoP.

## RESULTS

Results are presented in two phases: (1) Thematic findings from IDIs and (2) From qualitative findings to CoP design: A thematic mapping.

### Thematic Findings From IDIs

#### Participant Characteristics

Nineteen ECIs participated, with 58% female and 42% male, and 68% from sub-Saharan Africa and 32% from Latin America. Participants were predominantly doctoral-level investigators (PhDs [n = 6], MDs [n = 5], MD/PhDs [n = 2], and PharmD [n = 1]), with additional representation from master's (n = 4) and bachelor's (n = 1) investigators. Most were engaged in translational or population-based cancer research, with several reporting formal training in epidemiology, biostatistics, or clinical oncology.

#### Emerging Themes From Qualitative Analysis

Three themes emerged: (1) Institutional Capacity, (2) Mentorship, and (3) Aligning Research with Local Cancer Priorities. These themes reflect multilevel factors influencing ECI research careers. A detailed summary of these themes and associated subthemes is provided below, with deidentified quotes from participants to illustrate each theme.

##### Theme 1: Institutional capacity.

Participants frequently described structural constraints affecting their research trajectories. Limited access to infrastructure, including equipment, reagents, reliable internet, and administrative support, was commonly reported. Many also noted limited institutional prioritization of research and lack of protected time, particularly among clinicians balancing clinical and teaching responsibilities.

“*You can't just stop and walk away from patients. My research career wasn't going to grow… I wasn't going to get protected time*.”—Zambian faculty member.

Language barriers and limited access to research training further constrained career development, particularly in Francophone African and Latin American countries, as English proficiency is often required for publishing and grant applications.

“*The main barrier I see for my training here has been the language. Learning English as an adult has always been difficult. It's very important to handle English well*.”—Colombian PhD candidate.

Participants also described gaps in skill development, especially in grant writing, statistical analysis, and manuscript preparation, as well as limited access to formal research curricula.

Facilitators included international collaborations, early research exposure, and online learning opportunities. Prior research experience, institutional support, and professional networks were also identified as important enablers.

“*My colleagues, during my Master's, they're the ones that introduced me to the concept of research, so during my master's program, I had colleagues from Rwanda, and they were very active in research*.”—Tanzanian Oncologist.

##### Theme 2: Mentorship.

Mentorship emerged as a major determinant of research confidence and career development. Many participants reported limited access to high-quality mentorship and described hierarchical structures that constrained open communication. In addition, some noted that current mentors focused more on advancing their own careers, which limited the time they could dedicate to mentees.

“*I was interested in blood cancers, but not so many mentors in my country, especially in blood cancers. And mentorship here with research is more supervision (and less guidance and career support)*.”—Tanzanian Hematologist.

These dynamics often contributed to uncertainty in aligning their research activities with long-term career goals. When present, however, supportive mentors played a transformative role by providing guidance, facilitating access to opportunities, and integrating ECIs into ongoing research projects.

“*My mentor has exposed me to opportunities for grants, funding, or conferences that they have sent my way. They have also brought me into research that they have conceptualized and have sort of held my hand through that research so much that I can learn on the job and the work*.”—Nigerian Pathologist.

##### Theme 3: Aligning research with local cancer priorities.

Participants emphasized a need for research that reflects the cancer burdens, health system realities, and sociocultural contexts of their own regions. Many described a disconnect between local research needs and externally driven research agendas, particularly those shaped by HIC funders. This mismatch sometimes limited ECIs' ability to generate evidence that could directly influence patient care or health policies.

“*During my master’s research project, we had results that were completely different from high-income countries, and they are not explainable. We need to produce local research for local consumption…the clinical presentation is quite different, and the management we can offer is quite different*.”—Tanzanian Hematologist.

Barriers included limited access to data, funding, and stakeholder engagement.

“*I do need data and patient tissue samples. One of the fundamental problems in cancer research is finding an effective way to test potential therapies, and a lack of grants or funding from a grantor*.”—Malian Biologist

Furthermore, participants expressed a strong need for structured, tailored platforms that support collaboration, knowledge exchange, and development of contextually grounded research.

“*Creating a network will be a very good way forward because that will bring all of us together. Even if I get trained, I may end up getting lost somewhere if I'm just left on my own without any supportive network. Maybe through that, we can collaborate on projects that apply for grants, putting all our skills together for multidisciplinary cancer research*.”—Tanzanian Hematologist.

To complement and validate the IDI findings, facilitated discussions with ECIs outside the NCI GCRTP were conducted at the 2023 African Organization for Research and Training in Cancer (AORTIC) conference on the same topics covered in the interviews. The outcomes were consistent with all three themes, reinforcing the identified priorities.

### From Qualitative Findings to CoP Design: A Thematic Mapping

Qualitative findings directly informed the codesign of the virtual CoP (Table 1), linking identified needs to specific program features. These features are organized into a structured framework of three components—domain, community, and practice (Fig 2)—consistent with Wenger-Trayner's model.^35,36^ Design decisions were grounded in qualitative evidence and informed by ECIs' contextual expertise. The domain centers on cancer research, including research skill development, career development, and global collaboration. The community component fosters virtual engagement and networking through collaborative partnerships, research engagement, and peer relationships. The practice component supports learning resources and professional development, including training sessions, digital resources, and mentorship pathways.

**TABLE 1:** Mapping of Formative Qualitative Findings to Community of Practice Design Features

Identified Need From Qualitative Interviews	Illustrative Participant Concern	Corresponding CoP Design Feature
Limited institutional research infrastructure and protected time	Heavy clinical workload; insufficient research support	Virtual platform providing flexible access to training sessions and recorded content
Gaps in grant writing and research methodology skills	Limited formal training in proposal development and statistical analysis	Competency-focused workshops (eg, NIH grant process, manuscript development, research methods)
Variable access to high-quality mentorship	Mentorship perceived as supervisory rather than developmental	Cross-institutional mentorship engagement; inclusion of experienced investigators as invited speakers and discussion facilitators
Professional isolation within home institutions	Limited peer exchange and research networking opportunities	Structured peer networking sessions; breakout discussions; research spotlight presentations
Language barriers affecting engagement and publication	Challenges participating in English-dominant academic spaces	Inclusive facilitation practices; encouragement of multilingual engagement and subgroup discussions
Disconnect between external funding priorities and local research needs	Difficulty pursuing context-specific research agendas	Sessions from ECIs presenting funded research on context-specific research questions
Limited access to collaborative grant opportunities	Need for joint proposal development and multidisciplinary teams	Basecamp—platform for cross-country networking to foster collaborative grant development; share NIH grant writing resources
Need for a sustainable professional community	Risk of trainees becoming disconnected after formal training	Establishment of the ECI steering committee; charter development; shared governance model

Abbreviations: CoP, community of practice; ECIs, early-career investigators participating in the NCI Global Cancer Research Training Program based and working in LMICs; LMICs; low- and middle-income countries; NCI, National Cancer Institute; NIH, National Institutes of Health.

The steering committee guided the CoP development. They established an official charter with five components—(1) Purpose, (2) Membership and Governance, (3) Implementation, (4) Evaluation, and (5) Resource Sharing and Communication—and guiding principles, outlining core values and rules of engagement. They implemented an annual rotation of steering committee members, involving both US and LMIC participants, now in its second cohort. NCI/CGH staff coordinate logistics and facilitate connections with subject matter experts and organizations relevant to the community.

To expand engagement across settings, US and LMIC ECIs participating in the GCRTP and AORTIC 2023 were invited to join the CoP. At the time of manuscript preparation, the CoP included approximately 211 members, with the majority from LMICs.

To demonstrate how findings shaped early programming, the CoP held its inaugural session in April 2024, followed by seven additional sessions moderated by steering committee members, focused on community-identified priorities (Table 2). NCI/CGH staff established a digital platform (Basecamp) where members can access session recordings and training materials and engage in asynchronous discussions, addressing the need for flexible, accessible learning identified under the institutional capacity theme.

**TABLE 2:** Convened Sessions

Session Date	Session Topic	No. of Attendees
April 2024	Getting the funding that is right for you: Perspectives from Cancer Researchers in Africa	30
October 28, 2024	Hearing from Funders and their Awardees	59
February 25, 2025	Navigating the NIH Grant Application Process: K43 Grants	51
March 25, 2025	Navigating the NIH Grant Process: Post Submission Considerations	61
May 27, 2025	Navigating the NIH Grant Process: Post Award Considerations	33
June 24, 2025	Cancer Research Conversations: Cervical Cancer Screening Research and Capacity Building in Nigeria	42
July 29, 2025	Cancer Research Conversations: Breast Cancer Screening in Tanzania and Breast and Prostate Cancer Research in Ghana	46
September 30, 2025	Cancer Research Conversations: Research Advances and Lessons Learned—Mali and Burkina Faso	47

Abbreviation: NIH, National Institutes of Health.

CoP implementation is iterative, with continuous adaptations to member needs. An evaluation framework has been established to assess outputs and outcomes for future reporting.

## DISCUSSION

This study presents a formative qualitative analysis of ECIs' experiences in global cancer research careers. Three themes—limited institutional capacity, limited mentorship, and challenges aligning research with local priorities—were consistently identified and reflected multilevel barriers to research career development in LMIC settings. These findings are consistent with the prior literature.^37-45^

The study demonstrates how qualitative insights can inform the codesign of a CoP. By systematically linking identified needs to specific design features (Table 1), the approach provides a transparent, evidence-based framework for developing a tailored platform.^24-26,28-30^ The participatory process, which engaged ECIs in both data generation and design, supported alignment between community priorities and the CoP structure.

Early implementation observations suggest that the CoP design aligns with expressed needs. Across initial sessions, participation remained consistent, with strong engagement in topics such as grant writing and locally driven research, areas highlighted in the qualitative findings. These observations are descriptive and not intended as measures of effectiveness; a formal evaluation is ongoing.

A key strength of the CoP is its early focus on governance and sustainability. Establishing an ECI steering committee, adopting a community charter, and rotating steering committee members annually demonstrate a deliberate effort to share leadership and engage members as active architects of the community rather than passive recipients. This model ensures that leadership responsibilities and institutional knowledge are increasingly shared among investigators, strengthening the CoP's equity foundation over time. As the community grows beyond the initial training cohort, this structure will be vital to maintaining relevance and member ownership across diverse geographic and disciplinary contexts.

The virtual format, supported by a centralized digital platform, enables flexible participation and ongoing engagement, addressing barriers related to competing clinical and teaching responsibilities.^41,42^ In addition, the capacity to incorporate multilingual facilitation offers inclusive participation for Francophone African and Latin American members, an equity dimension that traditional in-person training models rarely address.^44,46^

While the CoP may address gaps in training, mentorship, and networking, it does not directly address structural institutional challenges such as limited research time, recognition of research productivity, or inadequate administrative support. Mentor-focused programming and the sharing of global best practices in career models could extend the CoP's influence into institutional environments.^47-51^ Future iterations of the CoP may engage institutional stakeholders to encourage broader systemic dialogue.

Several limitations of this study warrant consideration. Purposive sampling, while appropriate for qualitative analysis, might have introduced selection bias as participants were nominated by funded principal investigators and might have greater access to support. Findings reflect ECI perspectives only and do not include those of institutional leaders, mentors, or funders. Future studies should incorporate multilevel perspectives to examine systemic barriers and reforms. In addition, the CoP is in its early stages, and long-term outcomes, including sustained engagement and research productivity, have not yet been assessed. A formal evaluation framework has been established, and results will be reported in future work.

In conclusion, this study highlights the value of centering ECI perspectives in developing capacity-building initiatives for global cancer research. The codesign approach, grounded in qualitative evidence and guided by LMIC investigators themselves, produced a needs-driven platform with the potential to strengthen research training, foster collaboration, and support career development across settings. Supporting and investing in ECIs is essential; they are motivated and vital to advancing locally relevant cancer science with global impact.

## APPENDIX

**TABLE A1:** Predistributed Interview Questions to Participants

Question No.	Question Text
Question 1	Tell us about your career path—How did you get here?
Question 2	What barriers did you encounter (can be personal or professional)? What facilitators did you encounter (can be personal or professional)?
Question 3	What worked/is working for you at NCI under the Global Cancer Research Training Program?
Question 4	What did/is not working for you at NCI under the Global Cancer Research Training Program? What are the gaps/What is missing under the training program?
Question 5	What have you gained or hope to gain from this experience?
Question 6	Would you be interested in joining a virtual community of practice with other early-career cancer researchers, and if so, what would it look like? And what would you hope to gain from it?

NOTE. The following questions served as guiding prompts. Interviewers used probes and follow-up questions as needed based on participant responses.

Abbreviation: NCI, National Cancer Institute.
